# The prevalence of self-harm behavior in schizophrenia spectrum disorders: a systematic review and meta-analysis

**DOI:** 10.1192/j.eurpsy.2022.1978

**Published:** 2022-09-01

**Authors:** E. Lorentzen, O. Mors, J.N. Kjaer

**Affiliations:** Aarhus University Hospital, Dept. For Psychosis, Skejby, Denmark

**Keywords:** self-injury, schizophrénia, Prevalence, meta-analysis

## Abstract

**Introduction:**

Non-suicidal self-injury (NSSI) is intentional self-inflicted destruction of body tissue without suicidal intention, whereas deliberate self-harm (DSH) is self-inflicted destruction of body tissue regardless of intent. In clinical samples of patients with schizophrenia spectrum disorders (SSD) the estimates of prevalence and severity of self-harm behavior vary considerably.

**Objectives:**

The aim of this study is to investigate the prevalence of NSSI and DSH, respectively, in individuals with SSD.

**Methods:**

In adherence with PRISMA guidelines, a search of electronic databases (Pubmed, PsycInfo, and EMBASE) was conducted by two independent reviewers. Studies were included if the participants were diagnosed with SSD and DSH/NSSI were quantified by questionnaire or interview. Studies solely including patients with schizoaffective disorder, a severe intellectual disability, or autistic spectrum disorder were excluded. Meta-analysis of prevalence will be undertaken for NSSI and DSH, respectively. Further, the review will examine psychopathological correlates to DSH/NSSI, self-harming methods utilized in DSH/NSSI, and severity of DSH/NSSI.

**Results:**

Following duplicate removal, 1891 abstracts were initially identified through the database searches. 148 abstracts were included in screening of full-text articles of which 33 met the eligibility criteria. Nine authors were contacted for the purpose of obtaining additional data. Preliminary results found that the observed lifetime prevalence of NSSI ranged from 14.1% to 57.1%, whereas the observed lifetime prevalence of DSH ranged from 12.9% to 68.0%.

**Conclusions:**

Understanding how SSD and self-harming behavior are associated could identify subgroups of patients with SSD that are responsive to different pharmacological and psychosocial treatment approaches.

**Disclosure:**

No significant relationships.

